# Promoting Electrochemical Reversibility: Concave versus Convex Electrodes

**DOI:** 10.1021/acs.jpclett.5c00849

**Published:** 2025-04-20

**Authors:** Haotian Chen, Huanxin Li, Bedřich Smetana, Vlastimil Novák, Richard G. Compton

**Affiliations:** † Michigan Institute for Data and AI in Society, University of Michigan, 500 Church Street, Suite 600, Ann Arbor, Michigan 48109-1042, United States; ‡ Electrochemical Innovation Lab, Department of Chemical Engineering, University College London, London WC1E 7JE, United Kingdom; § Department of Chemistry and Physico-chemical processes, Faculty of Materials Science and Technology, VSB - Technical University of Ostrava, 17. listopadu 2172/15, 708 00 Ostrava-Poruba, Czech Republic; ∥ Department of Chemistry, Physical and Theoretical Chemistry Laboratory, Oxford University, South Parks Road, Oxford OX1 3QZ, Great Britain

## Abstract

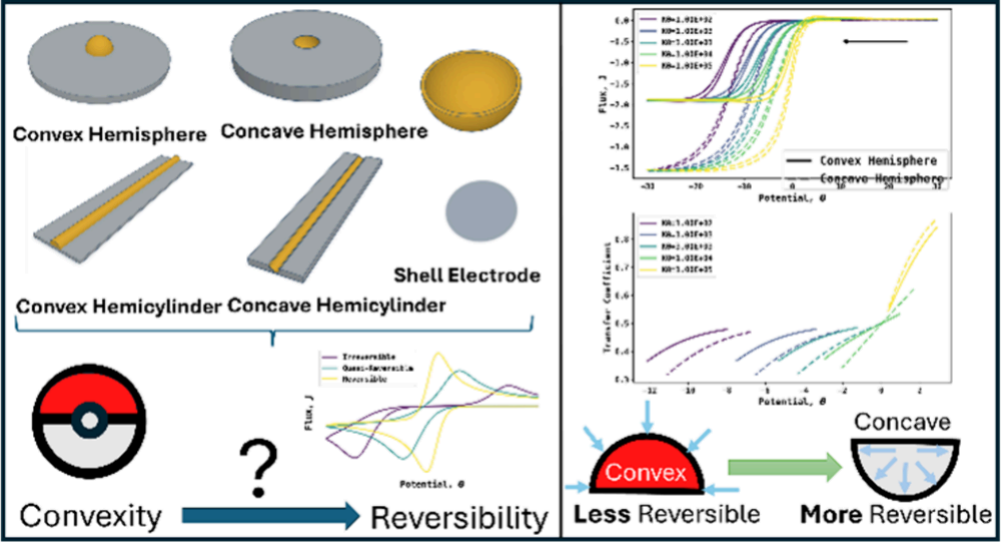

The importance of electrode shape, alongside electrode size, as key factors in controlling the reversibility or otherwise of electrochemical responses, is recognized at the microscopic level but is explored here via finite-element simulation on the macroscopic scale. Reduced overpotential is seen for concave surfaces relative to flat or convex surfaces, providing an unexplored avenue for the design and fabrication of electrodes, including composites for diverse applications where enhanced reversibility is desirable, such as in sensors and battery materials where the promotion of electrocatalytic responses is important.

The search for faster (more “reversible”) electrochemical reactions lies at the core of current research in the fields of both energy transformation and chemical sensing.^1−3^ In the former, the quest is for the maximization of electrical energy output from energy storage/generation systems and, in the case of batteries, the facilitation of easy recharging. In the latter, the reduction of overpotential allows the development of electrochemical sensors for diverse gases, pollutants, healthcare, etc., to proceed with the minimum chemical interference with the signal from the target species and the use of lower power devices. Overpotential, η, is the potential, *E*, above the thermodynamic equilibrium potential, (the Formal Potential, *E*_f_^0^) for the process of interest required to realize a desired electrochemical change:
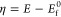
1Typically, the reversibility of an electrode process is identified via voltammetry—the measurement of current (*I*), as a function of the scanned potential (*E*) applied to the electrode of interest.^4,5^ If significant currents flow in opposite directions at potentials slightly different from the applied formal potential, then the system displays high electrochemical reversibility. Alternatively, if significant overpotential is required in both the cathodic and anodic directions, the process is irreversible. Figure 1 schematically shows the difference between reversible, quasi-reversible, and irreversible electrode kinetics for the case of the simple electrode reaction,
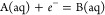
2in the form of a cyclic voltammogram in the dimensionless form, where the start potential and directions of sweeps are also shown.^6^*J* is the dimensionless flux, defined as *J* =  where *F* is the Faraday constant, *A* the area of the electrode, *c*_ref_^*^ the reference concentration (usually taken as 1 mol/m^3^), *D*_ref_ the reference diffusion coefficient (usually taken as 10^–9^ m^2^s^–1^), and *r*_*e*_ the radius (or a characteristic length) of the electrode. *J* is used to calculate the diffusion indicator, as shown in eq 5. The dimensionless potential θ is related to overpotential as θ = , where *R* is the gas constant, *T* the absolute temperature, and *E* the applied potential. The definition of all dimensionless parameters is given in the Supporting Information.

**Figure 1:**
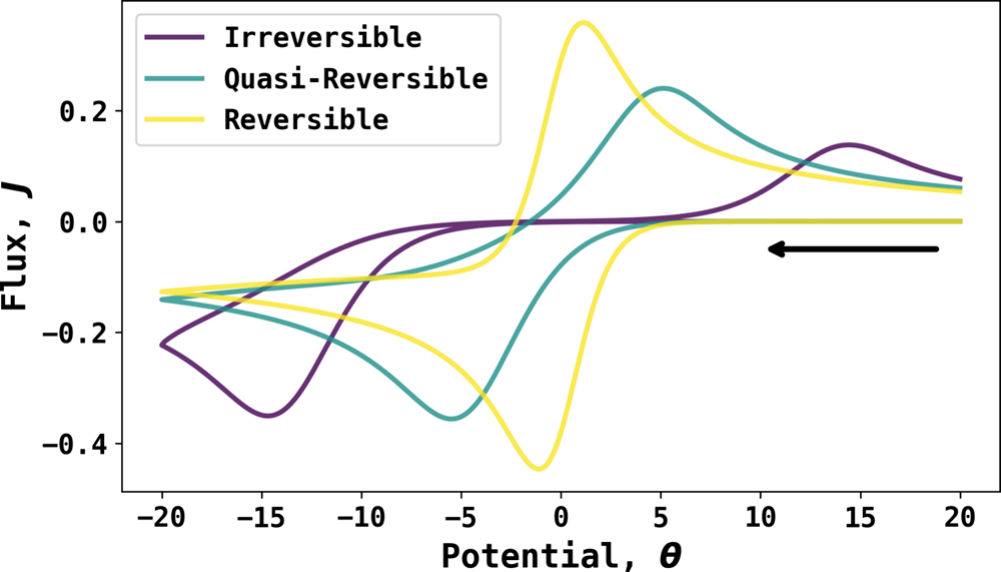
Simulated voltammograms from irreversible, quasi-reversible, and fully reversible electrochemical kinetics. The voltammograms are shown in dimensionless form, with the potential measured relative to the formal potential. The black arrow indicates the initial direction of the voltametric scan.

The limits of reversibility and irreversibility become clear from the definition of the transfer coefficients, α and β,^7,8^
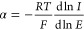
3
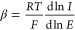
4derived from the analysis of experimental current–potential, are independent of any models of electron transfer (or of the overall number of electrons transferred). The voltammograms such as those in Figure 1 give a clear indication of electrochemical reversibility where, for the one electron conversion of A to B and the early, low current, part of the voltammogram, a value for α (for a reduction) or β (for an oxidation) approaching unity indicates a reversible process while values of ∼0.5 signal irreversibility.^9^ However, the form of the voltammogram reflects not only the electrode kinetics of the A/B redox couple but also the mass transport of the chemical species, A and B, to and from the electrode, which usually takes place predominantly or exclusively via diffusion. Consequentially, voltammograms in quiescent solution depend in part on the diffusional transport regimes experienced by different electrode geometries, with the contrasting transport modes becoming increasingly clear with a larger overpotential. This competition is well appreciated in the context of electrode size. Thus, microelectrodes, which possess at least one dimension on the scale of micrometers or less, experience more efficient diffusion than macroelectrodes which have sizes of the order of millimeters or more. This contrast arises since diffusive transport to macroelectrodes is essentially via planar (linear) diffusion whereas microelectrodes typically have to some greater or lesser extent, convergent diffusion (Figure 2). The voltammogram observed reflects a convolution of the electrode kinetics and the mass transport, resulting in a greater degree of reversibility being observed at larger electrodes. This manifests itself in a shift of the voltametric wave such that a larger overpotential is observed for smaller (micro)electrodes. Implicit in these observations is the fact that the reversibility of any redox process can reflect not only the identity of the participating chemical species but also the size of the electrode at which the reaction takes place.

**Figure 2:**
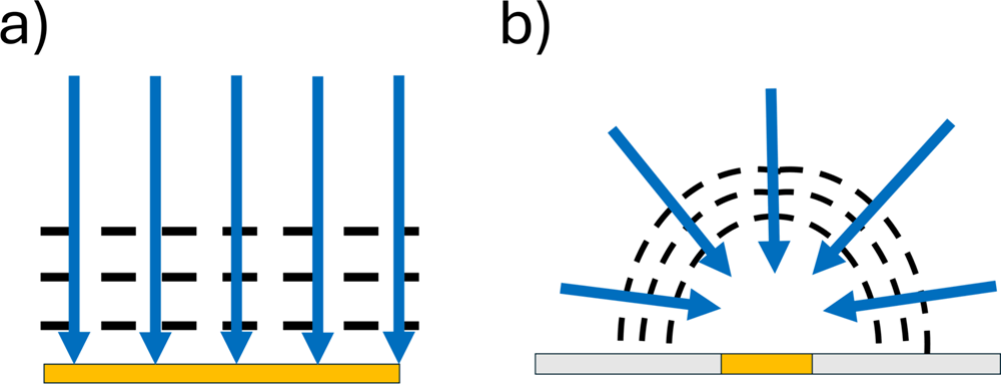
Schematic illustration of (a) linear diffusion at a macroelectrode and (b) convergent diffusion to a microelectrode.

The aim of the present paper is to explore the extent to which electrode *shape* influences observed electrochemical reversibility and specifically focus on contrasting flat, planar electrodes with those having significant curvature. This is a pertinent question since, apart from offering the potential for bespoke electrode design, many practical electrodes are composites made of particles of different shapes, often far from ideal geometries. Carbon particles can display diverse bent shapes, (including curvature-engineered graphene, nanotubes and fullerene) and all of which might have different voltametric signatures. Figure 3 shows, schematically, two particles namely a “broken” buckyball, C_60_, and a fractured carbon nanotube. The former might be approximated as a hemisphere, while the latter could be thought of as a hemicylinder.

**Figure 3:**
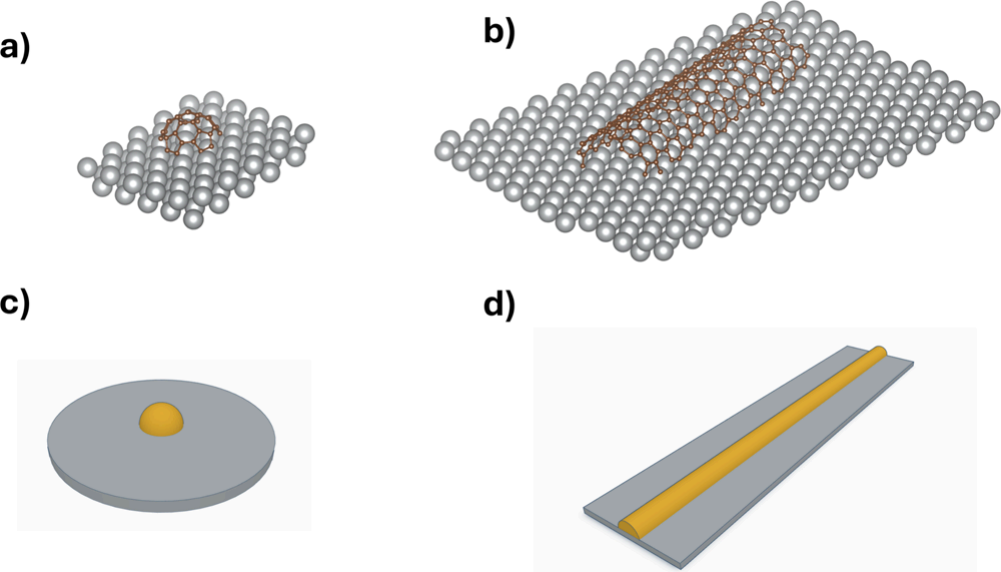
Schematic illustrations of (a) a broken C60 and (b) a fractured carbon nanotube on an atomically flat surface. The corresponding 3D geometry for (c) a convex hemisphere and (d) a convex hemicylinder electrode on a gray insulating surfaces.

Nanomaterials with concave/convex microstructures have gained significant attention due to their enhanced catalytic efficiencies across a wide range of major catalytical reactions. For example, solid carbon spheres exhibited excellent catalytic behavior of oxidative coupling of amines,^10^ gold nanoparticles increased catalytic rate of enzymatic cascades,^11^ and carbon nanotubes with appropriate surface curvature noticeably enhanced the efficiencies of nitrogen fixation,^12^ hydrogen evolution,^13^ and oxygen evolution/reduction.^14−16^ Curvature effect on catalytic efficiencies are attributed to multiple properties at the macroscopic scale including physical properties of fluids, and the microscopic scales correlating electronic structures, adsorption/desorption sites and surface energy.^17,18^ Despite the plethora of earlier literature investigating the microscopic effect of surface curvature,^19^ the macroscopic effect of curvature is largely neglected to the best of the authors’ knowledge. In this paper, the effect of macroscopic electrode geometries on observed electrochemical reversibility is investigated.

A specific question we seek to answer is whether a voltammogram measured on the inside surface of the hemisphere/hemicylinder is different from that on the outside surface. We hypothesize that voltammetry changes between electrodes with surfaces having concave and convex curvature, and if so, we ask to what extent does this show up in practical measurements? In particular, we consider the impact of shape in controlling mass transport and, hence, the observed electrochemical reversibility for concave and convex surfaces. We consider isolated structures rather than composites, but, in the latter context, we note the insightful studies of Kant and colleagues on both rough and fractal surfaces^20−32^ and note that the separation of mass transport from electrode kinetic effects generically allows the inference of intrinsic transfer coefficients.^31,32^ In the present work, we focus exclusively on the role of mass transport.

The mass transport to different types of electrodes can be usefully characterized by means of a diffusion indicator, DI, recently established by Le et al.^33−35^ to quantitatively characterize the extent to which the diffusion to the electrode is convergent (DI = 1) or linear (DI = 0). If the DI is below 0, it is an indicator of thin-layer diffusion. The diffusion indicator is defined as
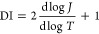
5where *J* and *T* are the dimensionless flux (proportional to current) at the electrode surface and the dimensionless time respectively, following a potential step from a potential at which no current flows to one where the flux is mass-transport-controlled. The dimensionless time is defined as *T* = , where *t* is time (in seconds). Oldham^36^ has generically considered the very short time chronoamperometric responses of electrodes of arbitrary shape enabling the corresponding calculation of the DI in some electrode geometries. The dimensionless diffusion layer thickness is √2*T*. To observe any curvature effect, the diffusion layer thickness should therefore be on the order of, or slightly larger than, the characteristic length of the electrode, i.e., √2*T* ≈ 1. In the following, we consider the full-time scaleup to steady state and note that the DI can be computed either for the entire electrode surface, or locally at specific area/points to reveal changes of diffusional regimes across an electrode surface. For example, the local diffusion indicator provided valuable insights into the complex diffusional behavior of non-uniform electrode geometries near the contact point of a sphere on a plane.^37^

In the following, we first simulate voltammetry of a simple one electron process at a hemisphere electrode where the electron transfer takes place either on the concave surface or on the convex side and compare it with what is seen at a planar flat disk electrode. The schematic illustration of a convex hemisphere is shown in Figure 3c. Simulations were made in two dimensions due to the cylindrical symmetry of the two systems. The Butler–Volmer equation is used to describe electrode kinetics when simulating voltammetry. The dimensionless Butler–Volmer equation is given as
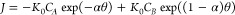
6where *K*_0_ is the dimensionless electrochemical rate constant, which is defined as −*K*_0_ = , note that *K*_0_ is general and does not apply to a specific electrode size. For example, considering an electrode size of 1 μm, a diffusion coefficient of 10^–9^ m^2^ s^–1^, an electrochemical rate constant of 10^–3^ m s^–1^, and *K*_0_ = 1. The range of experimental electrochemical rate constants encountered is usually below 0.1 m/s (10 cm/s) corresponding to *K*_0_ values of 10^2^ for a radius of 1 μm (a microelectrode of radius of 1 μm) and 10^5^ for a 1-mm-sized electrode (a macroelectrode of radius of 1 mm). *C*_A_ and *C*_B_ are dimensionless concentrations defined as for any species *j*, *C*_*j*_ = . When simulating voltammograms, the bulk concentrations of A and B are 1 and 0, respectively; α and β are fixed at 0.5 and *K*_0_ is varied.

To facilitate the comparison, we start by considering the diffusion indicator for the cases of interest. Figure 4a shows the (dimensionless) current–time response for the concave and convex surfaces of a hemisphere calculated using COMSOL (see the “Dimensionless Parameters and Equations” section in the Supporting Information), along with the corresponding response at a flat microdisk electrode resulting from a potential step from zero current to a fully diffusion-controlled regime. In the former cases, one or the other of the surfaces was active; the other was inactive (but see below for the case where both were simultaneously active). The radii of the disk and hemisphere were normalized to unity, and the flux was integrated across the electrode surface without normalization. In all three cases, at short time, DI tends to zero, whereas at long times, it intends to unity, showing the expected transition from planar diffusion at short times to convergent diffusion at long times. However, the change between limits occurs more gradually for the concave electrode and most rapidly for the convex electrode. The decreased transition rate of the concave surface reflects a contribution partly from thin layer behavior. The enhanced transition rate on the convex surface is due to a sooner development of convergent diffusion compared to a plane electrode. Given the differences revealed by DI modeling, we expect that this to be reflected in the current–voltage curves measured at the concave and convex electrode types.

**Figure 4:**
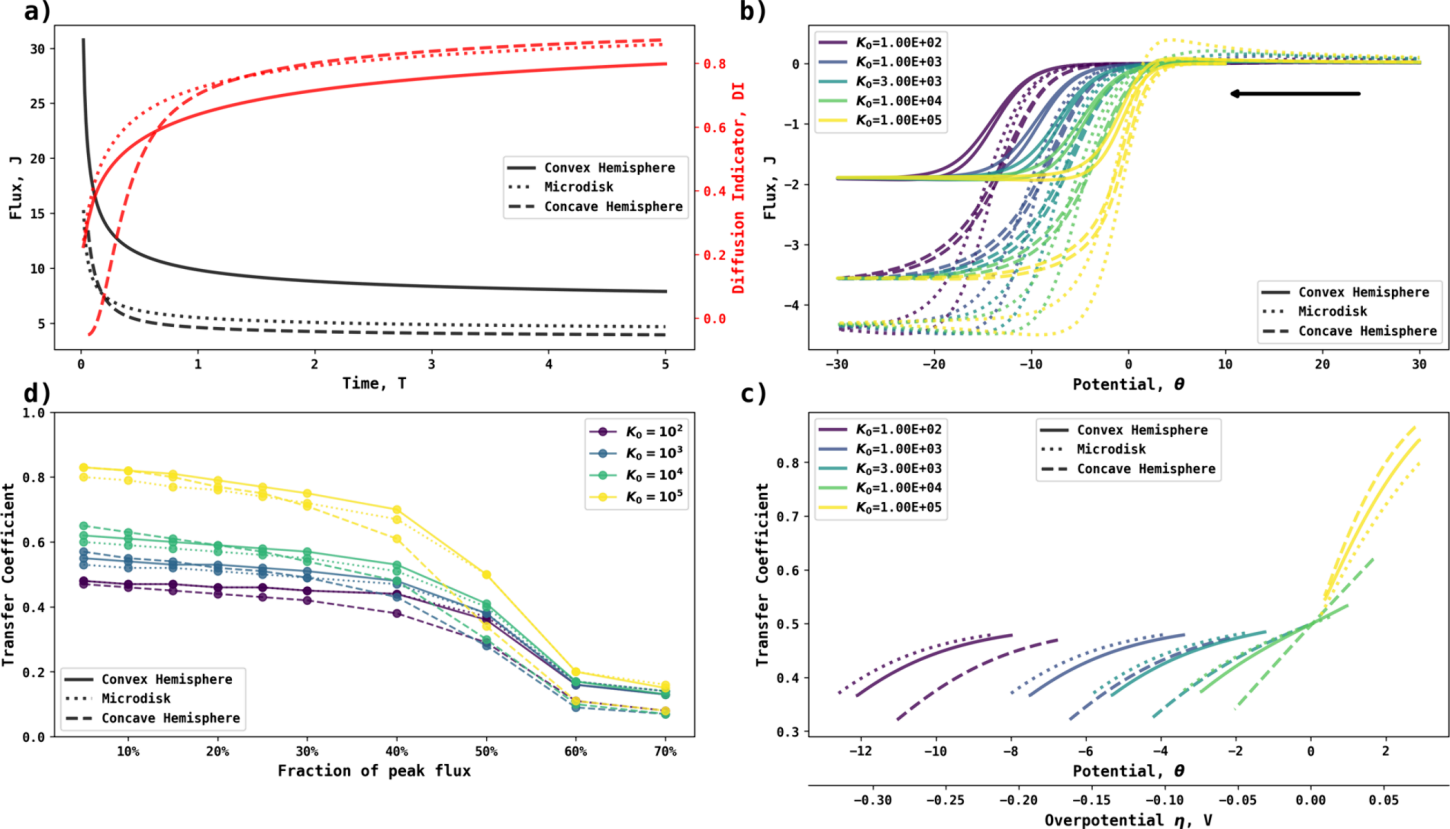
(a) Chronoamperogram (left axis) and diffusion indicator (right axis) at a convex hemisphere (solid line), a microdisk (dotted line), and a concave hemisphere (dashed line) electrode with radii of 1. (b) Cyclic voltammetry and (c) Tafel analysis at different electrochemical rate constants (10^2^ to 10^5^) and a dimensionless scan rate of 1. (d) Transfer coefficients at different fractions of peak fluxes. The black arrow indicates the initial direction of the voltammetric scan.

Figure 4b shows voltammograms simulated at various dimensionless heterogeneous rate constants for the surface of concave and convex hemispherical electrodes (of dimensionless radii unity) embedded in an insulating plane (Figure 3c) along with the equivalent voltammograms measured at a disk electrode of the same radius. The flux is the full (dimensionless) flux to the entire electrode (and hence reflects in part the different areas of the disk and hemispherical electrodes). Figure 4c shows the corresponding Tafel analysis inferred from the voltammograms, using eq 1. It is evident that the three electrode types display contrasting responses. We note that the voltammograms measured at the convex hemisphere appear at a higher overpotential than the concave electrode. They show an apparent greater level of irreversibility for a fixed value of the electrochemical rate constant. The microdisk electrode reveals intermediate behavior. This observation is consistent with the inferences made based on the DI.

In the case of Figure 4c, the increased overpotential is evident since, in the cases simulated, the formal potential is assumed and therefore known. In the analysis of experimental data this is often not the case for many systems of interest. In this situation, the experimenter is likely to rely on the use of Tafel analysis to compare different electrodes. Figure 4d shows that measurements of the transfer coefficient measured at different fractions of the maximum current observed in the voltammogram show significant differences between the electrode types increasingly at larger currents where the influence of mass transport is greater.

The different voltametric signatures along with their Tafel analysis are consistent with the hypothesis that concave and convex surfaces show different responses, with the former displaying a greater degree of apparent reversibility. To further validate this idea, we consider next a hemicylindrical electrode, as shown in Figure 3d, of the dimensionless radius of unity and make analogous simulations as for the hemispherical electrodes considering both concave and convex surfaces together with, for comparison, a flat microband electrode.

The data for the diffusion indicator and the voltammetry are shown in Figure 5, and the details of the model and simulation are given in the “COMSOL Simulation” section in the Supporting Information. The results for the transfer coefficients for different curvatures are shown in Figure 5d. The results show the difficulty of using a single measurement of the transfer coefficient in the absence of any knowledge of the formal potential or the standard electrochemical rate constant; both are frequently unknown. The trends shown reflect multiple factors including the transition toward electrochemical reversibility as the electrochemical rate constant increased, the effect of increasing current density which desensitizes the current response toward potential and again the shape of the electrode with significant differences seen between concave, convex, and flat surfaces with all other parameters fixed. These data therefore confirm the idea that electrode shape controls apparent electrochemical reversibility as much as the electrode size.

**Figure 5:**
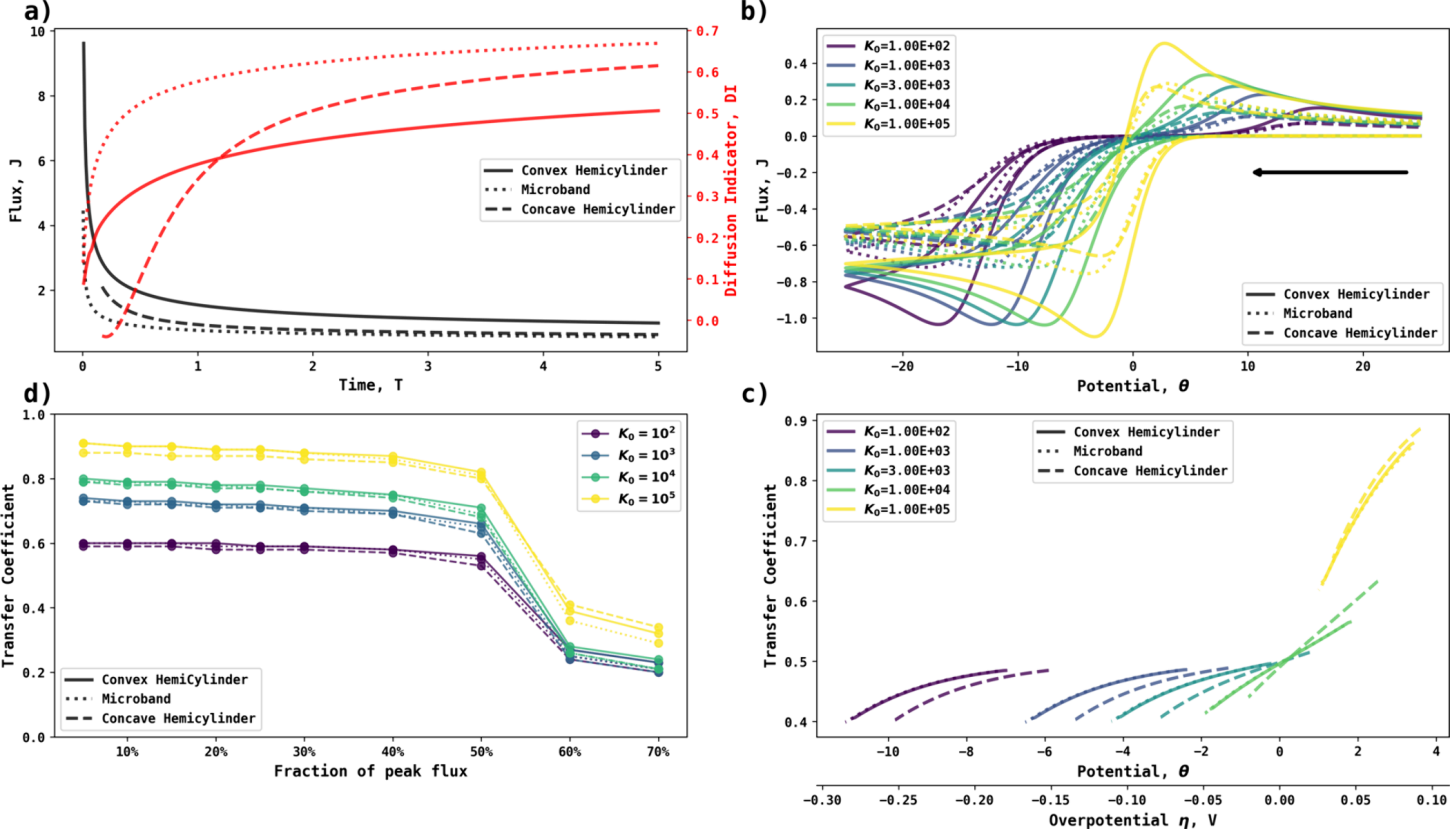
(a) Chronoamperogram (left) and diffusion indicator(right) at the convex hemicylinder (solid), microband (dashed), and concave hemicylinder (dashed) surface with radius of 1. (b) Cyclic voltammetry and (c) Tafel analysis at different electrochemical rate constants (10^2^ to 10^5^) and a dimensionless scan rate of 1. (d) Transfer coefficients at different fractions of peak fluxes. The black arrow indicates the initial direction of the voltammetric scan.

As a further example, we consider a free-standing “shell electrode”, as shown schematically in Figure 6a, considering the voltammetry measured for the electrode in bulk solution with both the inner, concave surface and the outer convex surface simultaneously active. Figure 7 shows the chronoamperometry, DI analysis, voltammetry, and Tafel analysis of the entire shell electrode together with the contributions from the inner and outer surfaces. As is anticipated from the response for the convex and concave hemispheres reported above, the onset of electrolysis occurs at lower potentials for the inner surface. This is evident from the concentration profile shown in Figures 6b and 6c, showing the extent of concentration depletion as a function of potential, where the quasi-thin layer response from the concave surface predominates at shorter times and hence earlier in the voltammetric scan. This observation emphasizes the contrasting electrochemical responses seen for electrodes of different curvatures. The transfer coefficients at different fractions of peak fluxes are shown in Figure 7d, showing that the concave shell surface enhances electrochemical reversibility more than its convex counterpart when the electrochemical reaction is (quasi)irreversible, as evidenced by its higher transfer coefficients.

**Figure 6:**
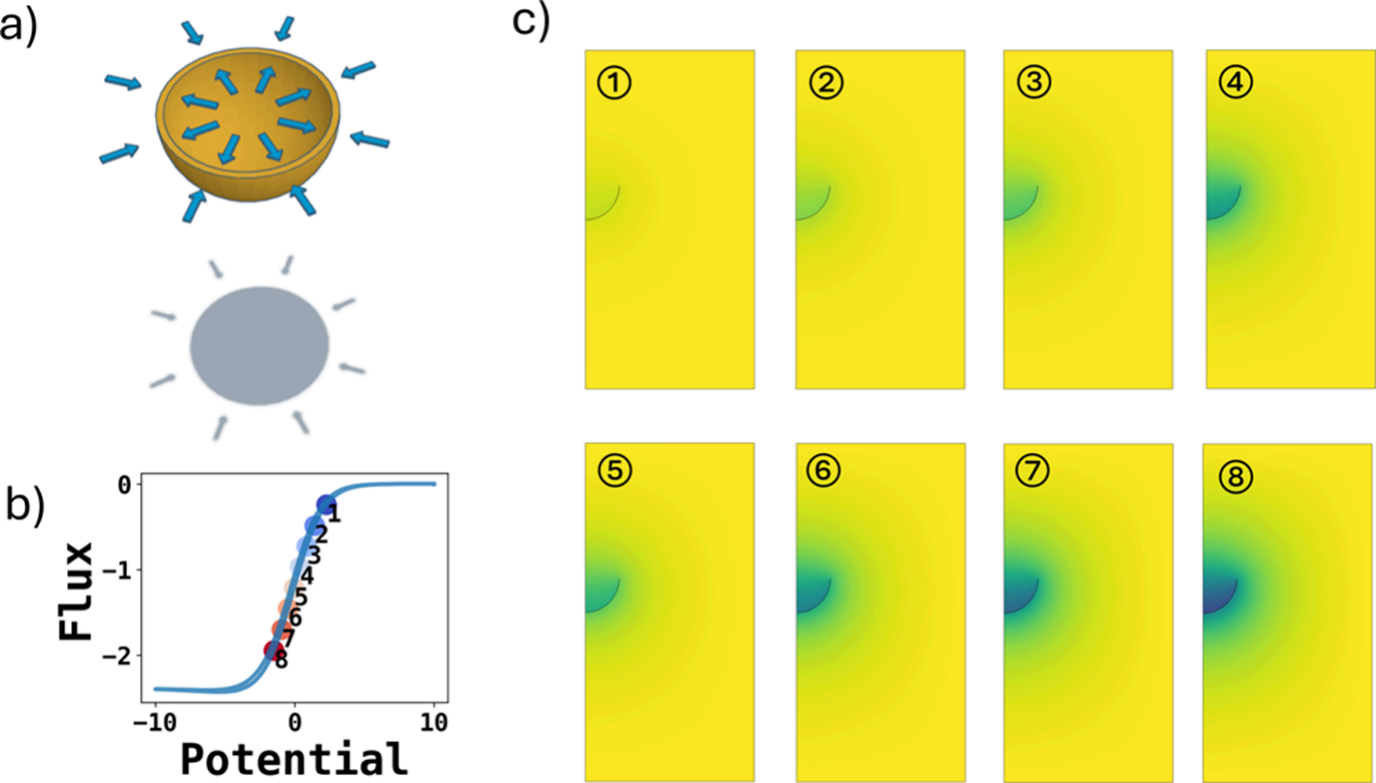
(a) Schematic illustration of a shell electrode in solution with both the inner, concave surface and the outer, convex surface electrochemically active. The thickness of the shell is negligible. (b) Voltammetry at the concave surface, while the convex surface is simultaneously active at a dimensionless scan rate of 0.1 and *K*_0_ = 10^5^. The scatter points represent the 10% to 80% forward scan peak flux. (c) Concentration profile at 10% to 80% peak flux at the scatter of the voltammogram shown in panel (b).

**Figure 7:**
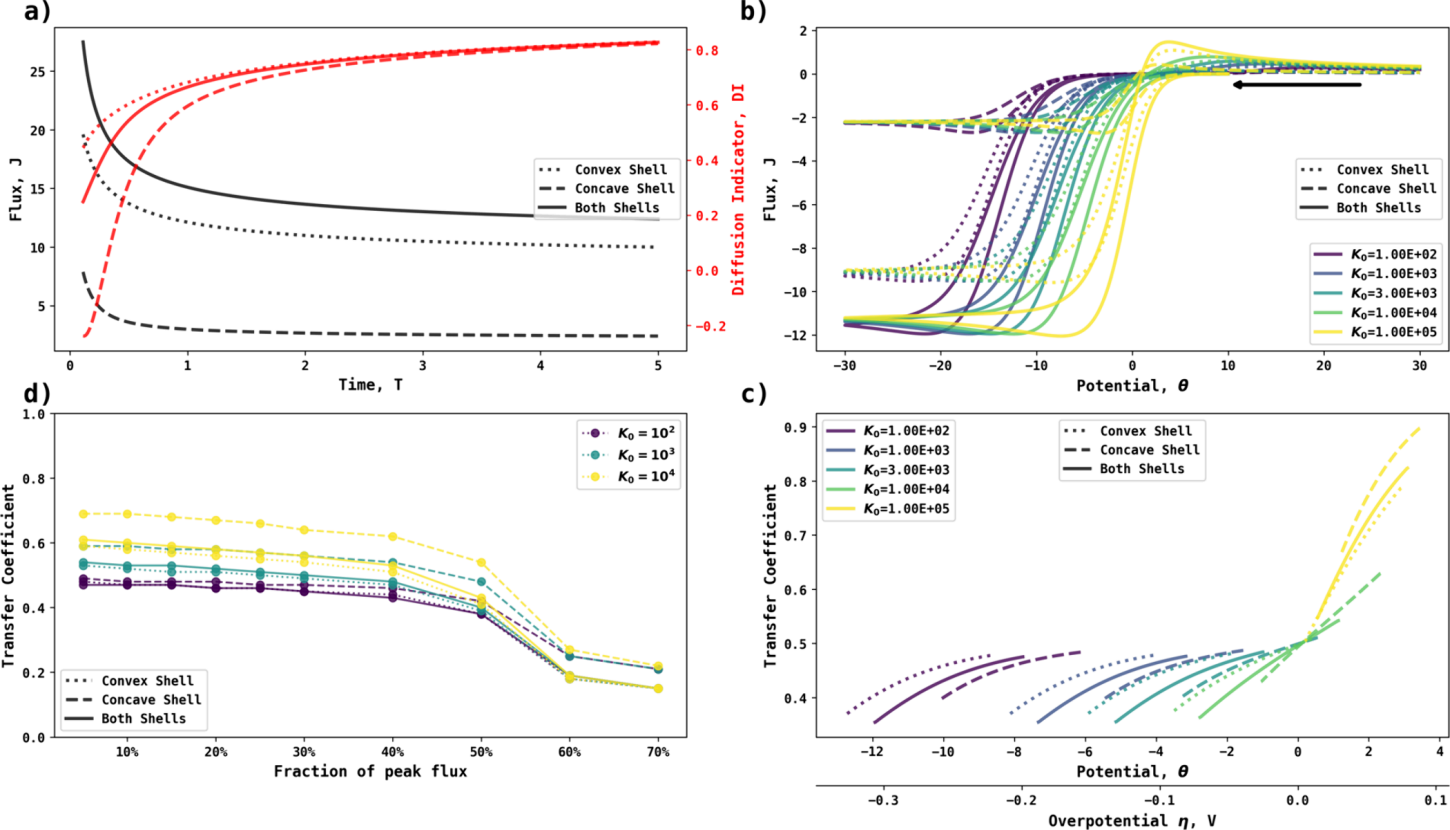
(a) Chronoamperogram (left) and diffusion indicator (right) at convex (dotted line) and concave (dashed line) surfaces of a shell electrode with radii of 1. (b) Cyclic voltammetry and (c) Tafel analysis at a dimensionless scan rate of 1. (d) Transfer coefficients at different fractions of peak fluxes. The black arrow indicates the initial direction of voltammetric scan.

In conclusion, we have shown that electrode shape influences the reversibility of electrochemical responses as much as electrode size. Thus, the presence of concave features on an electrode encourages enhanced reversibility, whereas convex structures promote relative irreversibility. These concepts can underpin the design of bespoke free-standing electrodes/nanomaterials while also providing a rationale in part for the diversity of responses seen for electrode composites formed from conductive particles of different shapes. Equally the inference of “reversibility” when comparing different electrodes or electrode materials purely and simply because of Tafel slopes without reference to the pertinent formal potential is likely to mislead and discard otherwise attractive materials. At the same time, the exploration of particle shape as much as particle size is encouraged particularly in light of simulations showing the interplay of mass transport with electrode kinetics for differently shaped diffusion fields. The study of macroscopic properties may shed new light on understanding the curvature effect.

## Methods

Simulations were performed using COMSOL Multiphysics 6.2 with electroanalysis module.^38^ A 2D model was constructed to simulate a hemicylinder electrode, and a 2D axis-symmetric model was constructed for hemisphere and shell electrodes. The fundamental mass transport equations are the 2D diffusion equation and Butler–Volmer equation for electrode kinetics. The meshing is mostly refined at the electrode surface to capture concentration gradients with a maximum elemental size of 0.01. The simulation was solved by using a time-dependent solver with a backward differentiation formula (BDF) solver. Note that the use of COMSOL in constrained geometries is challenging with respect to adequate meshing and achieving good convergence. The results in the main text should be seen as semiquantitative with the purpose of revealing the qualitative differences between concave and convex surfaces.

## Supporting Information Available

Dimensionless parameters and equations; COMSOL Simulation; transfer coefficients and fraction of peak currents (PDF)

## References

[ref1] Braun A. Electrochemical Energy Systems: Foundations, Energy Storage and Conversion; Walter de Gruyter GmbH & Co KG, 2018.

[ref2] Ugo P.; Marafini P.; Meneghello M. Bioanalytical Chemistry; De Gruyter, 2021, 10.1515/9783110589160.

[ref3] Zhang X.; Ju H.; Wang J. Electrochemical Sensors, Biosensors and Their Biomedical Applications; Academic Press, 2011.

[ref4] Compton R. G.; Banks C. E. Understanding Voltammetry, 4th Edition; World Scientific, 2025, 10.1142/q0471.

[ref5] Compton R. G.; Sanders G. H. W.; Yang J. M. Electrode Potentials; Oxford University Press, 2025.

[ref6] Compton R. G.; Laborda E.; Kaetelhoen E.; Ward K. R. Understanding Voltammetry: Simulation of Electrode Processes; World Scientific, 2020.

[ref7] Guidelli R.; Compton R. G.; Feliu J. M.; Gileadi E.; Lipkowski J.; Schmickler W.; Trasatti S. Defining the transfer coefficient in electrochemistry: An assessment (IUPAC Technical Report). Pure Appl. Chem. 2014, 86 (2), 245–258. 10.1515/pac-2014-5026.

[ref8] Guidelli R.; Compton R. G.; Feliu J. M.; Gileadi E.; Lipkowski J.; Schmickler W.; Trasatti S. Definition of the transfer coefficient in electrochemistry (IUPAC Recommendations 2014). Pure Appl. Chem. 2014, 86 (2), 259–262. 10.1515/pac-2014-5025.

[ref9] Li D.; Lin C.; Batchelor-McAuley C.; Chen L.; Compton R. G. Tafel analysis in practice. J. Electroanal. Chem. 2018, 826, 117–124. 10.1016/j.jelechem.2018.08.018.

[ref10] Qiao Z.; Cao H.; Wang J.; Yang H.; Yao W.; Wang J.; Cheetham A. K. Curvature-Induced Electron Spin Catalysis with Carbon Spheres. Angew. Chem. Int. Ed. 2025, 64 (1), e202412745 10.1002/anie.202412745. 39218803

[ref11] Díaz S. A.; Choo P.; Oh E.; Susumu K.; Klein W. P.; Walper S. A.; Hastman D. A.; Odom T. W.; Medintz I. L. Gold Nanoparticle Templating Increases the Catalytic Rate of an Amylase, Maltase, and Glucokinase Multienzyme Cascade through Substrate Channeling Independent of Surface Curvature. ACS Catal. 2021, 11 (2), 627–638. 10.1021/acscatal.0c03602.

[ref12] Gao F.-Y.; Hu S.-J.; Zhang X.-L.; Zheng Y.-R.; Wang H.-J.; Niu Z.-Z.; Yang P.-P.; Bao R.-C.; Ma T.; Dang Z.; et al. High-Curvature Transition-Metal Chalcogenide Nanostructures with a Pronounced Proximity Effect Enable Fast and Selective CO2 Electroreduction. Angew. Chem. Int. Ed. 2020, 59 (22), 8706–8712. 10.1002/anie.201912348. 31884699

[ref13] Zheng X.; Zhang G.; Xu X.; Liu L.; Zhang J.; Xu Q. Synergistic effect of mechanical strain and interfacial-chemical interaction for stable 1T-WSe2 by carbon nanotube and cobalt. Appl. Surf. Sci. 2019, 496, 143694 10.1016/j.apsusc.2019.143694.

[ref14] She F.; Guo Z.; Liu F.; Yu Z.; Chen J.; Fan Y.; Lei Y.; Chen Y.; Li H.; Wei L. Curvature-Dependent Electrochemical Hydrogen Peroxide Synthesis Performance of Oxidized Carbon Nanotubes. ACS Catal. 2024, 14 (14), 10928–10938. 10.1021/acscatal.4c01637.

[ref15] Ma N.; Zhang Y.; Wang Y.; Zhao J.; Liang B.; Xiong Y.; Luo S.; Huang C.; Fan J. Curvature effects regulate the catalytic activity of Co@N4-doped carbon nanotubes as bifunctional ORR/OER catalysts. J. Colloid Interface Sci. 2024, 654, 1458–1468. 10.1016/j.jcis.2023.10.115. 37924660

[ref16] Sui H.; Guo Q.; Xiang M.; Kong X.; Zhang J.; Ding S.; Su Y. Theoretical Insights of Curvature Effects of FeN4-Doped Carbon Nanotubes on ORR Activity. J. Phys. Chem. Lett. 2024, 15 (32), 8257–8264. 10.1021/acs.jpclett.4c01932. 39106043

[ref17] Serp P.; Castillejos E. Catalysis in Carbon Nanotubes. ChemCatChem. 2010, 2 (1), 41–47. 10.1002/cctc.200900283.

[ref18] Ouyang G.; Wang C. X.; Yang G. W. Surface Energy of Nanostructural Materials with Negative Curvature and Related Size Effects. Chem. Rev. 2009, 109 (9), 4221–4247. 10.1021/cr900055f. 19670888

[ref19] Wen H.; Zhao Z.; Luo Z.; Wang C. Unraveling the Impact of Curvature on Electrocatalytic Performance of Carbon Materials: A State-of-the-Art Review. ChemSusChem 2024, 17 (10), e202301859 10.1002/cssc.202301859. 38246873

[ref20] Kant R. Can current transients be affected by the morphology of the nonfractal electrode?. Phys. Rev. Lett. 1993, 70 (26), 4094–4097. 10.1103/PhysRevLett.70.4094. 10054045

[ref21] Kant R. Can one electrochemically measure the statistical morphology of a rough electrode?. J. Phys. Chem. 1994, 98 (6), 1663–1667. 10.1021/j100057a020.

[ref22] Kant R.; Rangarajan S. Effect of surface roughness on diffusion-limited charge transfer. J. Electroanal. Chem. 1994, 368 (1-2), 1–21. 10.1016/0022-0728(93)03069-2.

[ref23] Kant R.; Rangarajan S. K. Diffusion to rough interfaces: finite charge transfer rates. J. Electroanal. Chem. 1995, 396 (1), 285–301. 10.1016/0022-0728(95)03971-I.

[ref24] Kant R. Diffusion-limited reaction rates on self-affine fractals. J. Phys. Chem. B 1997, 101 (19), 3781–3787. 10.1021/jp963141p.

[ref25] Kant R.; Rangarajan S. Effect of surface roughness on interfacial reaction–diffusion admittance. J. Electroanal. Chem. 2003, 552, 141–151. 10.1016/S0022-0728(03)00039-1.

[ref26] Kant R.; Jha S. K. Theory of anomalous diffusive reaction rates on realistic self-affine fractals. J. Phys. Chem. C 2007, 111 (38), 14040–14044. 10.1021/jp075525t.

[ref27] Rama K.; Shweta D.; Jasmin K. Electrode disorder, electrochemical processes and governing length scales. J. Indian Inst. Sci. 2016, 96 (4), 365–382.

[ref28] Kumar R.; Kant R. Experimental corroboration of general phenomenological theory for dynamics of EDL in viscous medium on rough heterogeneous electrode. Electrochim. Acta 2017, 257, 473–482. 10.1016/j.electacta.2017.10.046.

[ref29] Kant R.; Goel H. In Situ Electrochemical Impedance Spectroscopic Method for Determination of Surface Roughness and Morphological Convexity. J. Phys. Chem. Lett. 2021, 12 (41), 10025–10033. 10.1021/acs.jpclett.1c02935. 34622659

[ref30] Kant R.; Mishra G. K. Theory for Heterogeneous Electron Transfer Kinetics on Nanocorrugated Atomic Stepped Metal Electrodes. J. Phys. Chem. C 2023, 127 (14), 6884–6899. 10.1021/acs.jpcc.2c08690.

[ref31] Mishra G. K.; Kant R. Semi-microscopic Theory for the Current Rectification Phenomenon in Nanogap Molecular Devices. J. Phys. Chem. A 2023, 127 (13), 3048–3062. 10.1021/acs.jpca.3c00332. 36974459

[ref32] Neha; Kant R. Theory for Outer Sphere Electron Transfer Coupled with Ion Transfer Kinetics on Atomically Stepped Metal. J. Phys. Chem. C 2024, 128 (30), 12399–12413. 10.1021/acs.jpcc.4c02297.

[ref33] Le H.; Kätelhön E.; Compton R. G. Characterising the nature of diffusion via a new indicator: Microcylinder and microring electrodes. J. Electroanal. Chem. 2019, 855, 113602 10.1016/j.jelechem.2019.113602.

[ref34] Le H.; Compton R. G. Comparative chronoamperometry: Spheres, discs, cylinders and bands. J. Electroanal. Chem. 2020, 866, 114149 10.1016/j.jelechem.2020.114149.

[ref35] Usha Rani R.; Rajendran L. Diffusion indicator for hemispheroidal and ring ultramicroelectrode geometries for E and EC′ reactions. Electrochem. Commun. 2021, 128, 107071 10.1016/j.elecom.2021.107071.

[ref36] Oldham K. B. The short-time chronoamperometric behaviour of an electrode of arbitrary shape. J. Electroanal. Chem. Interfacial Electrochem. 1991, 297 (2), 317–348. 10.1016/0022-0728(91)80032-L.

[ref37] Elliott J. R.; Compton R. G. Local diffusion indicators: A new tool for analysis of electrochemical mass transport. J. Electroanal. Chem. 2022, 908, 116114 10.1016/j.jelechem.2022.116114.

[ref38] Introduction to Comsol multiphysics. In COMSOL Multiphysics, Burlington, MA, 1998; p 32. Accessed February 1998.

