# Supplementary material for: Promoting Electrochemical Reversibility: Concave versus Convex Electrodes
Source: J Phys Chem Lett. 2025 Apr 20;16(17):4189–95. doi: 10.1021/acs.jpclett.5c00849 (PMC12051189; doi:10.1021/acs.jpclett.5c00849)
Supplement: Supplementary file 1 — jz5c00849_si_001.pdf [file jz5c00849_si_001.pdf]

Supporting Information for “Promoting Electrochemical Reversibility: Concave versus Convex Electrodes”

Haotian Chen[1], Huanxin Li[2,4], Bedřich Smetana[3], Vlastimil Novák[3], Richard G. Compton[4*]

[1] Michigan Institute for Data and AI in Society, University of Michigan, Ann Arbor, 500 Church Street, Suite 600, Ann Arbor, MI 48109-1042, United States

[2] Electrochemical Innovation Lab, Department of Chemical Engineering, University College London, London WC1E 7JE, United Kingdom

[3] Department of Chemistry and Physico-chemical processes, Faculty of Materials Science and Technology, VSB - Technical University of Ostrava, 17. listopadu 2172/15, 708 00 Ostrava-Poruba, Czech Republic

[4] Department of Chemistry, Physical and Theoretical Chemistry Laboratory, Oxford University, South Parks Road, Oxford OX1 3QZ, Great Britain

* Corresponding author.

Email address: Richard.compton@chem.ox.ac.uk (R. G. Compton)

Contents

1 Dimensionless Parameters and Equations....2
2 COMSOL Simulation....3
2.1 Geometry Generation....3
2.2 Mesh Generation and Time Stepping....4
2.3 Convergence Tests....6
2.4 Electrochemistry Boundary Conditions....7
2.5 Calculating Flux....7
3 Transfer Coefficients at Fractions of Peak Currents....8
3.1 Convex Hemisphere Electrode....8
3.2 Concave Hemisphere Electrode....8
3.3 Convex Hemicylinder Electrode....9
3.4 Concave Hemicylinder Electrode....9
3.5 Microband Electrode....10
3.6 Microdisk Electrode....10

3.7 Convex Shell Electrode 10
3.8 Concave Shell Electrode 11
3.9 Shell Electrode with Both Surfaces Active 11
4 References 12

1 Dimensionless Parameters and Equations

Although COMSOL supports simulations in SI units, simulations in this paper are carried out in dimensionless form for its enhanced numerical stability and generalizability.[1, 2] The dimensionless parameters are defined in Table S1.

Table S1. Definitions of dimensionless parameters. R,F,T are the Gas Constant, Faraday Constant and Temperature, respectively. Elsewhere T also denotes dimensionless time whilst R is also a polar coordinate. r_e is the radius of electrode. $D_{ref} = 10^{-9}\ m^2 s^{-1}$ and $c^*_{ref} = 1M$.

Parameter	Definition
Concentration	$C_j = \frac{c_j}{c^*_{ref}}$
Diffusion coefficient	$d_j = \frac{D_j}{D_{ref}}$
Spatial Coordinates (Cartesian)	$X = \frac{x}{r_e}, Y = \frac{y}{r_e}, Z = \frac{z}{r_e}$
Spatial Coordinates (Polar)	$R = \frac{r}{r_e}, Z = \frac{z}{r_e}$
Time	$T = \frac{D_{ref} t}{r_e^2}$
Overpotential	$\theta = \left(\frac{F}{RT}\right)\left(E - E_f^0\right)$
Scan rate	$\sigma = \left(\frac{r_e^2}{D_{ref}}\right)\left(\frac{F}{RT}\right)\nu$
Current	$J = \frac{I}{\frac{FAc^*_{ref} D_{ref}}{r_e}}$
Electrochemical rate constant	$K_0 = \frac{k_0 r_e}{D_{ref}}$

The dimensionless diffusion equation in the Cartesian 2D model used for hemicylinder simulation is:

$$\frac{\partial C_j}{\partial T} = d_j\left(\frac{\partial^2 C_j}{\partial X^2} + \frac{\partial^2 C_j}{\partial Y^2}\right)$$

The dimensionless diffusion equation in 2D axisymmetric model for the hemisphere and shell electrode is:

$$\frac{\partial C_j}{\partial T} = d_j\left(\frac{\partial^2 C_j}{\partial R^2} + \frac{1}{R}\frac{\partial C}{\partial R} + \frac{\partial^2 C_j}{\partial Z^2}\right)$$

In the symmetry boundary or electrode surface boundary, the no flux boundary condition is:

$$-\hat{\boldsymbol{n}} \cdot \left(-d_j \nabla C_j\right) = 0$$

where $\hat{\boldsymbol{n}}$ is the outward pointing normal vector and ∇ is the Laplacian operator.

For the outer boundary of simulation, analyte concentration is unperturbed by the electrochemical reaction at the electrode surface. The bulk concentration is simply:

$C_j = C_j^*$where C_j^* is the bulk concentration of the analyte.

The electrochemistry boundary condition is given in section 2.4.

2 COMSOL Simulation

As an example, the details and implementation of the COMSOL Multiphysics[3] 6.2 simulation for a convex hemicylinder electrode and a convex hemisphere electrode are given below.

2.1 Geometry Generation

Because the convex hemicylinder is infinitely long and axial symmetrical, simulation is reduced to 2D and only half of a convex hemicylinder is simulated. The simulation geometry for a convex hemicylinder electrode is illustrated in Figure S1 and a convex hemisphere electrode is illustrated in Figure S2.There are two simulation domains: the inner ordinary simulation domain and the outer infinite element simulation domain. The dimensionless radius of hemicylinder is 1, and the width of simulation domain was determined from Brownian motion. The width of the inner domain is:

$w_{max} = 4\sqrt{d_{ref}T_{sim}}$and the width of the outer infinite element domain is $1.2w_{max}$. T_{sim} is the length scale of simulation. For cyclic voltammetry with a single cycle, T_{sim} is:

$T_{sim} = 2\,\frac{abs(\theta_i - \theta_v)}{\sigma}$where θ_i and θ_v are the initial and vertex potential, respectively.

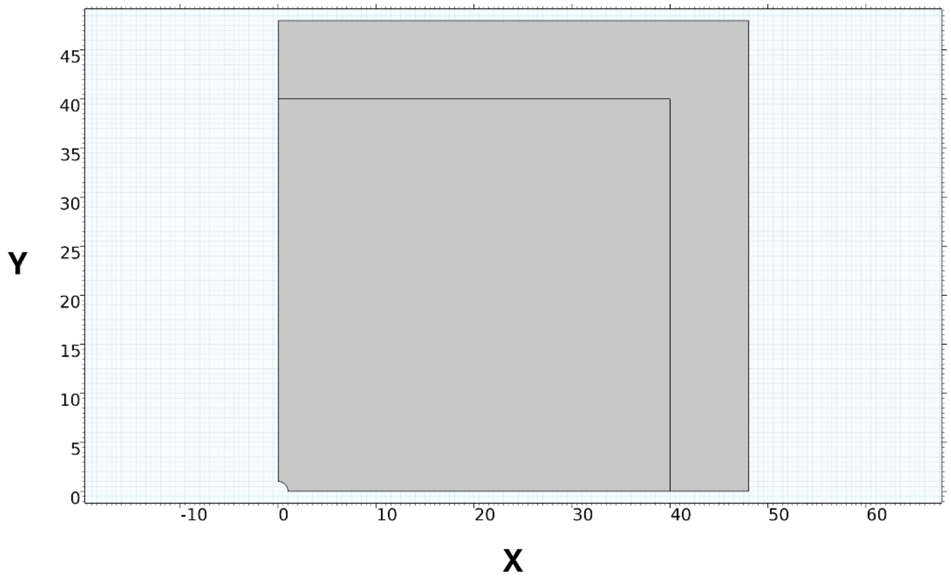

Figure S1. The simulation geometry for a convex hemicylinder electrode.

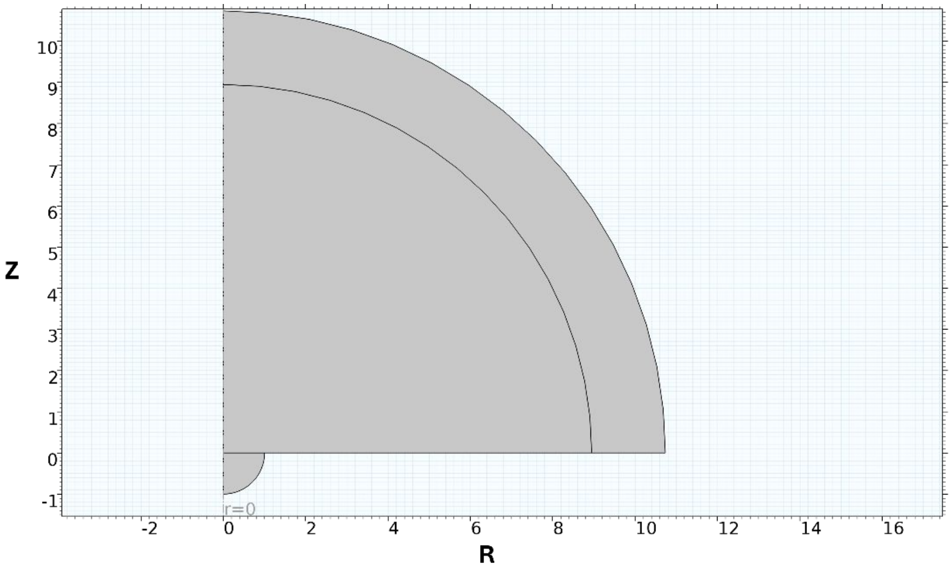

Figure S2. The simulation geometry for a concave hemisphere electrode.

2.2 Mesh Generation and Time Stepping

Two forms of meshes were generated; a free triangular mesh for the inner domain and a mapped mesh for the outer infinite element domain. For the inner domain, the mesh was set to “Extra fine”. The element size was based on the electrode boundary with a

maximum element size of 0.01. The generated mesh for concave cylinder is shown in Figure S3 with a total of 7555 elements. The generated mesh for concave hemisphere is shown in Figure S4 with a total of 10566 elements. The generated mesh is denser near the electrode surface, which aligns with the principle of electrochemical reaction and the steeper concentration gradient near the electrode surface.

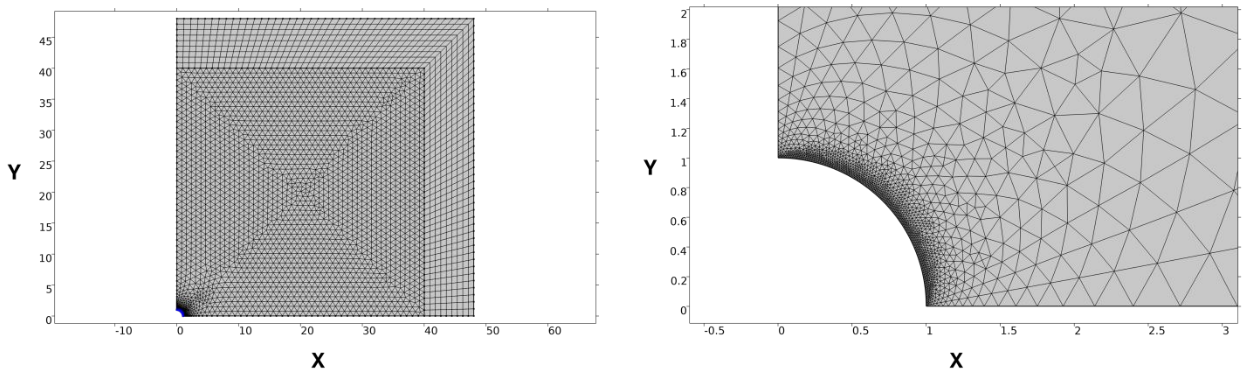

Figure S3. (left) The generated mesh for a convex hemicylinder electrode and (right) a closer look at the mesh near the electrode surface.

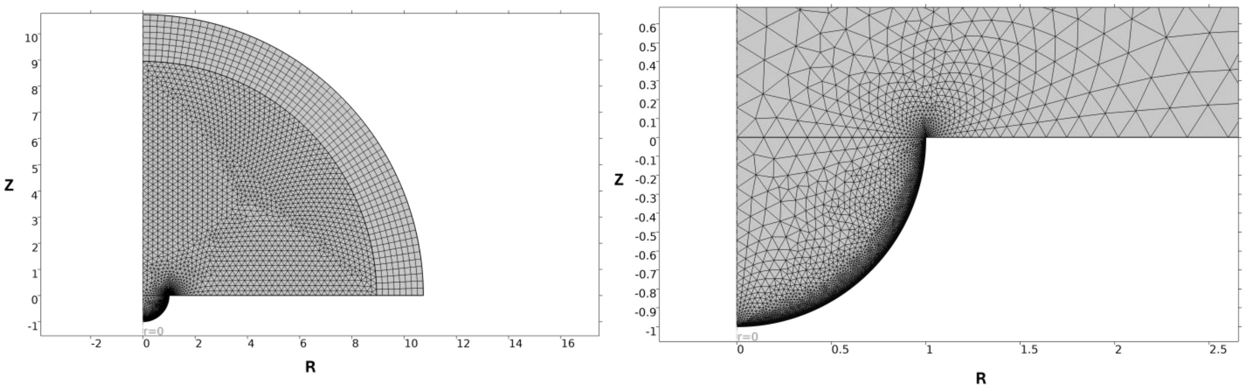

Figure S4. (left)The generated mesh for concave hemisphere electrode and (right) a closer look at the mesh near the electrode surface. Note the more refined mesh at the intersection of the concave hemisphere with the insulating plane.

For simulation of chronoamperometry, the time step size is determined by the potential step size, and the time step size is determined to be:

$$\Delta T = \frac{\Delta\theta}{\sigma}$$

Selecting an optimal set of spatial and temporal elements size is vital for the success of simulation. An element size too small costs significantly more time and memory, while an element size too large gives inaccurate results. Thus, it is very important to perform convergence tests to find the optimal sets of spatial and temporal parameters (ΔX and $\Delta \theta$) to balance accuracy and cost. The convergence tests are reported in the next section.

2.3 Convergence Tests

Convergence tests in finite element simulations for ensuring the accuracy and reliability of results. Two of the more important simulation parameters, ΔX and $\Delta\theta$, corresponding to spatial and temporal refinement respectively. At a dimensionless scan rate of 1 and a concave hemicylinder electrode, voltammetry at a reversible ($K_0 = 10^5$) and irreversible ($K_0 = 10^2$) electrode kinetics are simulated. The forward scan peak fluxes and forward scan peak potential are examined to ensure the model is satisfactorily converged. Figure S5 shows the convergence test on ΔX tested from 10^{-3} to 5×10^{-2} and Figure S6 shows the convergence test on $\Delta\theta$ tested from 10^{-2} to 10^{-1}. Both figures suggested that the simulation parameters used ($\Delta X = 10^{-2}$ and $\Delta\theta = 10^{-2}$) are sufficiently small and the simulation results, including the overall voltammograms, peak fluxes and peak potentials, are insensitive to the meshing parameters selected. Time stepping scheme is set to "intermediate" for both voltammetry and chronoamperometry experiments. With "intermediate" time stepping, the solver takes an additional step with each interval.

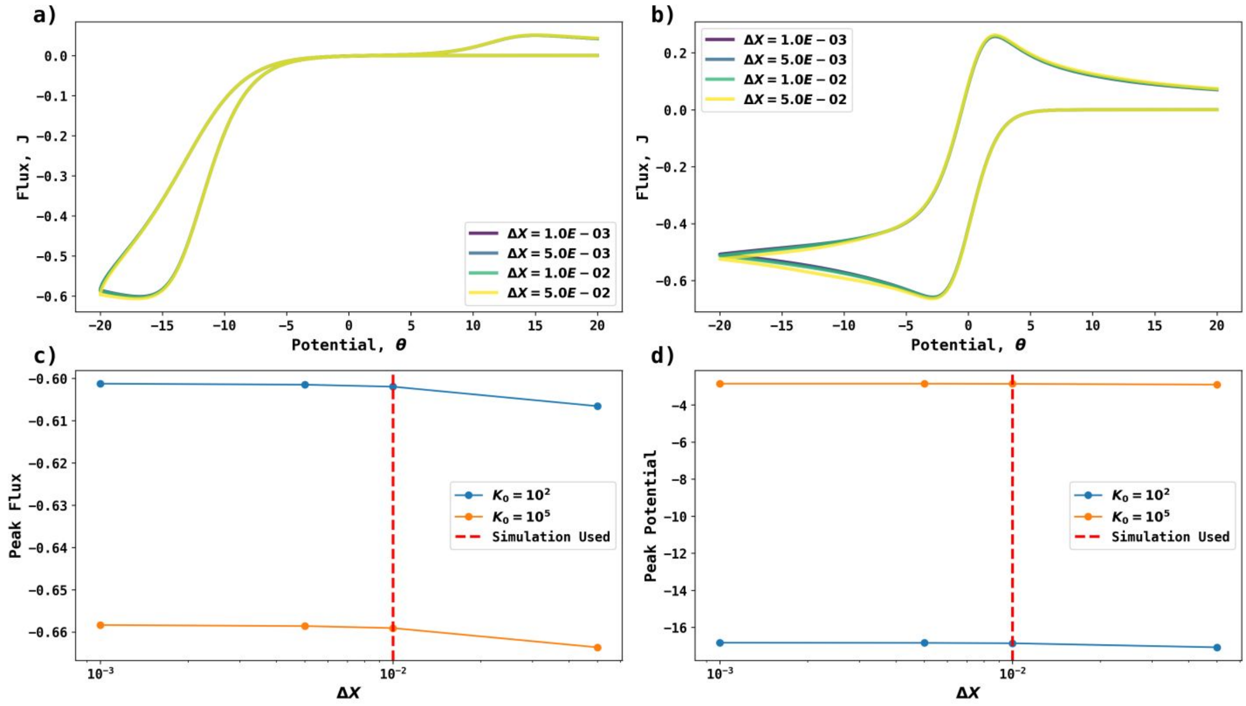

Figure S5. Convergence test on ΔX, the maximum element size on voltammetry with a dimensionless scan rate of 1 and $\Delta\theta = 10^{-2}$. (a) Voltammograms with different ΔX at $K_0 = 10^2$. (b) (a) Voltammograms with different ΔX at $K_0 = 10^5$. (c) Peak flux and (d) peak potential as a function of ΔX.

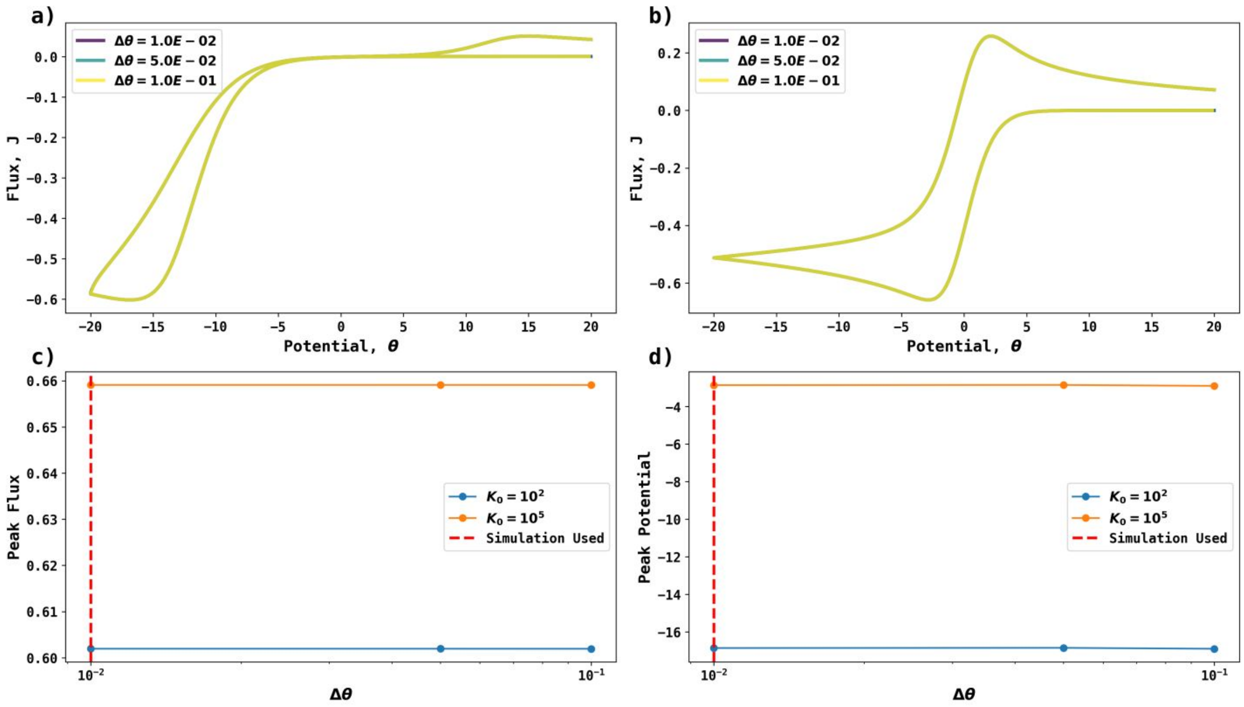

Figure S6. Convergence test on $\Delta\theta$, the potential step size, on voltammetry with a dimensionless scan rate of 1 and $\Delta X = 10^{-2}$. (a) Voltammograms with different $\Delta\theta$ at $K_0 = 10^2$. (b) (a) Voltammograms with different $\Delta\theta$ at $K_0 = 10^5$. (c) Peak flux and (d) peak potential as a function of $\Delta\theta$.

2.4 Electrochemistry Boundary Conditions

The electrochemistry boundary conditions for cyclic voltammetry simulations were made assuming that the electrode kinetics follows the Butler-Volmer equation which in dimensionless form is:

$J = -K_0 C_A \exp(-\alpha\theta) + K_0 C_B \exp((1-\alpha)\theta)$where C_a and C_b are the species in the one electron redox couple and α is the reductive transfer coefficient. α was set to 0.5 for all simulations reported.

The boundary conditions for chronoamperometry are simply setting the concentration on electrode surface to zero at the start of potential step.

2.5 Calculating Flux

After completion of time stepping, the flux to the electrode surface is calculated by the integration:

$$J = -d_{ref} \int_{\theta=0°}^{\theta=90°} \hat{\boldsymbol{n}} \cdot \vec{\mathbf{J}}\, d\theta$$ where $\hat{\boldsymbol{n}}$ is the normal vector and $\vec{\mathbf{J}}$ is the flux vector calculated by COMSOL.

3 Transfer Coefficients at Fractions of Peak Currents

The following sections provide tables of apparent transfer coefficients calculated using Equation 3 in the text for different electrode geometries at different combinations of fractions of peak current and electrochemical rate constants, K_0.

3.1 Convex Hemisphere Electrode

Table S2. Apparent transfer coefficient measured at different electrochemical rate constants and fraction of peak current for convex hemisphere electrode.

fraction of peak current	1.00E+02	1.00E+03	3.00E+03	1.00E+04	1.00E+05
5%	0.48	0.5	0.55	0.62	0.83
10%	0.47	0.5	0.54	0.61	0.82
15%	0.47	0.49	0.53	0.6	0.81
20%	0.46	0.49	0.53	0.59	0.79
25%	0.46	0.48	0.52	0.58	0.77
30%	0.45	0.47	0.51	0.57	0.75
40%	0.44	0.45	0.48	0.53	0.7
50%	0.36	0.36	0.38	0.41	0.5
60%	0.16	0.16	0.16	0.17	0.2
70%	0.13	0.13	0.13	0.13	0.15

3.2 Concave Hemisphere Electrode

Table S3. Apparent transfer coefficient measured at different electrochemical rate constants and fraction of peak current for concave hemisphere electrode.

fraction of peak current	1.00E+02	1.00E+03	3.00E+03	1.00E+04	1.00E+05
5%	0.47	0.51	0.57	0.65	0.83
10%	0.46	0.5	0.55	0.63	0.82
15%	0.45	0.49	0.54	0.61	0.8
20%	0.44	0.48	0.52	0.59	0.77
25%	0.43	0.47	0.51	0.57	0.75

30%	0.42	0.45	0.49	0.54	0.71
40%	0.38	0.4	0.43	0.48	0.61
50%	0.29	0.27	0.28	0.3	0.34
60%	0.11	0.09	0.09	0.1	0.11
70%	0.08	0.07	0.07	0.07	0.08

3.3 Convex Hemicylinder Electrode

Table S4. Apparent transfer coefficient measured at different electrochemical rate constants and fraction of peak current for convex hemicylinder electrode.

fraction of peak current	1.00E+02	1.00E+03	3.00E+03	1.00E+04	1.00E+05
5%	0.6	0.69	0.74	0.8	0.91
10%	0.6	0.68	0.73	0.79	0.9
15%	0.6	0.68	0.73	0.79	0.9
20%	0.6	0.68	0.72	0.78	0.89
25%	0.59	0.67	0.72	0.78	0.89
30%	0.59	0.67	0.71	0.77	0.88
40%	0.58	0.66	0.7	0.75	0.87
50%	0.56	0.62	0.66	0.71	0.82
60%	0.27	0.27	0.27	0.28	0.39
70%	0.23	0.23	0.23	0.24	0.32

3.4 Concave Hemicylinder Electrode

Table S5. Apparent transfer coefficient measured at different electrochemical rate constants and fraction of peak current for concave hemicylinder electrode.

fraction of peak current	1.00E+02	1.00E+03	3.00E+03	1.00E+04		1.00E+05
5%	0.59	0.68	0.73	0.79		0.88
10%	0.59	0.67	0.72	0.78		0.88
15%	0.59	0.67	0.72	0.78		0.87
20%	0.58	0.67	0.71	0.77		0.87
25%	0.58	0.66	0.71	0.77		0.87
30%	0.58	0.66	0.7	0.76		0.86
40%	0.57	0.64	0.69	0.74		0.85
50%	0.53	0.6	0.63	0.68		0.8
60%	0.24	0.24	0.24	0.26		0.41
70%	0.2	0.2	0.2	0.21		0.34

3.5 Microband Electrode

Table S6. Apparent transfer coefficient measured at different electrochemical rate constants and fraction of peak current for microband electrode.

fraction of peak current	1.00E+02	1.00E+03	3.00E+03	1.00E+04	1.00E+05
5%	0.6	0.68	0.73	0.79	0.91
10%	0.6	0.68	0.73	0.79	0.9
15%	0.6	0.68	0.72	0.78	0.9
20%	0.59	0.67	0.72	0.78	0.89
25%	0.59	0.67	0.71	0.77	0.89
30%	0.59	0.66	0.71	0.76	0.88
40%	0.58	0.65	0.69	0.75	0.86
50%	0.55	0.61	0.65	0.69	0.81
60%	0.25	0.25	0.25	0.26	0.36
70%	0.21	0.21	0.21	0.21	0.29

3.6 Microdisk Electrode

Table S7. Apparent transfer coefficient measured at different electrochemical rate constants and fraction of peak current for microdisk electrode.

fraction of peak current	1.00E+02	1.00E+03	3.00E+03	1.00E+04	1.00E+05
5%	0.48	0.5	0.53	0.6	0.8
10%	0.47	0.49	0.52	0.59	0.79
15%	0.47	0.49	0.52	0.58	0.77
20%	0.46	0.48	0.51	0.57	0.76
25%	0.46	0.47	0.5	0.56	0.74
30%	0.45	0.47	0.49	0.55	0.72
40%	0.44	0.45	0.47	0.51	0.67
50%	0.37	0.37	0.38	0.4	0.5
60%	0.17	0.17	0.17	0.17	0.2
70%	0.14	0.14	0.14	0.14	0.16

3.7 Convex Shell Electrode

Table S8. Apparent transfer coefficient measured at different electrochemical rate constants and fraction of peak current for convex shell electrode.

fraction of peak current	1.00E+02	1.00E+03	3.00E+03	1.00E+04	1.00E+05

5%	0.48	0.49	0.53	0.59	0.79
10%	0.47	0.49	0.52	0.58	0.78
15%	0.47	0.48	0.51	0.57	0.77
20%	0.46	0.48	0.51	0.56	0.75
25%	0.46	0.47	0.5	0.55	0.74
30%	0.45	0.47	0.49	0.54	0.72
40%	0.44	0.45	0.47	0.51	0.67
50%	0.38	0.38	0.39	0.41	0.52
60%	0.18	0.18	0.18	0.18	0.22
70%	0.15	0.15	0.15	0.15	0.17

3.8 Concave Shell Electrode

Table S9. Apparent transfer coefficient measured at different electrochemical rate constants and fraction of peak current for concave shell electrode.

fraction of peak current	1.00E+02	1.00E+03	3.00E+03	1.00E+04	1.00E+05
5%	0.59	0.68	0.73	0.79	0.88
10%	0.59	0.67	0.72	0.78	0.88
15%	0.59	0.67	0.72	0.78	0.87
20%	0.58	0.67	0.71	0.77	0.87
25%	0.58	0.66	0.71	0.77	0.87
30%	0.58	0.66	0.7	0.76	0.86
40%	0.57	0.64	0.69	0.74	0.85
50%	0.53	0.6	0.63	0.68	0.8
60%	0.24	0.24	0.24	0.26	0.41
70%	0.2	0.2	0.2	0.21	0.34

3.9 Shell Electrode with Both Surfaces Active

Table S10. Apparent transfer coefficient measured at different electrochemical rate constants and fraction of peak current for combining the concave and convex surfaces of the shell electrode.

fraction of peak current	1.00E+02	1.00E+03	3.00E+03	1.00E+04	1.00E+05
5%	0.47	0.5	0.54	0.61	0.82
10%	0.47	0.5	0.53	0.6	0.81
15%	0.47	0.49	0.53	0.59	0.8

20%	0.46	0.48	0.52	0.58	0.78
25%	0.46	0.48	0.51	0.57	0.77
30%	0.45	0.47	0.5	0.56	0.75
40%	0.43	0.45	0.48	0.53	0.7
50%	0.38	0.38	0.4	0.43	0.54
60%	0.19	0.19	0.19	0.19	0.24
70%	0.15	0.15	0.15	0.15	0.19

4 References

(1) Compton, R. G.; Banks, C. E. *Understanding Voltammetry 4th Edition*; World Scientific, 2025. DOI: doi:10.1142/q0471.

(2) Compton, R. G.; Laborda, E.; Kaetelhoen, E.; Ward, K. R. *Understanding voltammetry: simulation of electrode processes*; World Scientific 2020.

(3) Multiphysics, C. Introduction to Comsol multiphysics®. *COMSOL Multiphysics, Burlington, MA, accessed Feb* **1998**, *9* (2018), 32.
